# Sustainment stories: a qualitative analysis of barriers to sustainment of the National Rural Transitions of Care Nurse Program

**DOI:** 10.1186/s12913-021-07420-1

**Published:** 2022-01-28

**Authors:** Mary Ava Nunnery, Heather Gilmartin, Michaela McCarthy, Lexus Ujano-De Motta, Ashlea Wills, Lynette Kelley, Christine D. Jones, Chelsea Leonard

**Affiliations:** 1 Denver/Seattle Center of Innovation for Veteran-Centered and Value Driven Care, VA Eastern Colorado Healthcare System, 1700 N. Wheeling St, Aurora, CO 80045 USA; 2 Health Systems, Management and Policy, University of Colorado, School of Public Health, Aurora, CO 80045 USA; 3 University of Colorado Anschutz Medical Campus, Division of Hospital Medicine, Department of Medicine, 12401 E 17th Place, Aurora, CO 80045 USA

**Keywords:** Care coordination, Rural healthcare, Veterans health administration, Implementation science, Sustainment, Qualitative research, Veterans, Barriers

## Abstract

**Background:**

Understanding how to successfully sustain evidence-based care coordination interventions across diverse settings is critical to ensure that patients continue to receive high quality care even after grant funding ends. The Transitions Nurse Program (TNP) is a national intervention in the Veterans Administration (VA) that coordinates care for high risk veterans transitioning from acute care VA medical centers (VAMCs) to home. As part of TNP, a VA facility receives funding for a full-time nurse to implement TNP, however, this funding ends after implementation. In this qualitative study we describe which elements of TNP sites planned to sustain as funding concluded, as well as perceived barriers to sustainment.

**Methods:**

TNP was implemented between 2016 and 2020 at eleven VA medical centers. Three years of funding was provided to each site to support hiring of staff, implementation and evaluation of the program. At the conclusion of funding, each site determined if they would sustain components or the entirety of the program. Prior to the end of funding at each site, we conducted midline and exit interviews with Transitions nurses and site champions to assess plans for sustainment and perceived barriers to sustainment. Interviews were analyzed using iterative, team-based inductive deductive content analysis to identify themes related to planned sustainment and perceived barriers to sustainment.

**Results:**

None of the 11 sites planned to sustain TNP in its original format, though many of the medical centers anticipated offering components of the program, such as follow up calls after discharge to rural areas, documented warm hand off to PACT team, and designating a team member as responsible for patient rural discharge follow up. We identified three themes related to perceived sustainability. These included: 1) Program outcomes that address leadership priorities are necessary for sustainment.; 2) Local perceptions of the need for TNP or redundancy of TNP impacted perceived sustainability; and 3) Lack of leadership buy-in, changing leadership priorities, and leadership turnover are perceived barriers to sustainment.

**Conclusions:**

Understanding perceived sustainability is critical to continuing high quality care coordination interventions after funding ends. Our findings suggest that sustainment of care coordination interventions requires an in-depth understanding of the facility needs and local leadership priorities, and that building adaptable programs that continually engage key stakeholders is essential.

**Supplementary Information:**

The online version contains supplementary material available at 10.1186/s12913-021-07420-1.

## Background

Sustainment of health care programs is an important topic in the field of implementation science. Care coordination interventions are uniquely challenging to sustain since they often involve creating a new position, which is expensive. A widely accepted definition of sustainability is “‘ongoing stage of use’ after implementation” [1], and Campbell et al. suggest that a program’s ability to leave a lasting impact is the real defining mark of successful implementation [2]. However, because each healthcare setting has a unique context with differences in leadership, system level factors, and infrastructure, it can be difficult to predict where a program will be sustained [3]. Thus, there is little guidance on how to ensure sustainment of successful programs for few studies address which factors impact long-term sustainability [3].

A variety of factors from system level barriers to relationships between staff may play into a program’s life and survival in unique settings [4]. With limited time, resources, and funding available to support implementation of evidence-based healthcare programs, sustainment is critical to continuation of successful outcomes [5]. Factors that may influence sustainment include program design, differences in facilitation throughout implementation, organizational characteristics, and the community environment [2]. The complexity of the intervention may also influence how likely it is to be adopted and therefore sustained [6].

Due to this limited understanding of how to sustain care coordination interventions, our team sought to better understand barriers to planned sustainment of the Transitions Nurse Program (TNP) at 11 VA medical centers across the country. TNP was developed to address the unique needs of the rural veteran population [7]. After a pilot that showed enrolled veterans were significantly more likely to have a follow-up visit within 14 days of discharge and trends toward reductions in unplanned 30-day readmissions, TNP was funded by VA’s Office of Rural Health (ORH) for national dissemination [7, 8]. TNP is a care coordination intervention that provides transitional care for high-risk rural Veterans who are hospitalized at an urban VA Medical Center (VAMC) and return to rural VA primary care settings [8]. Participating VAMCs were provided with information that could be used to support sustainment through audit and feedback, such as site level program outcomes data as well as visual representations of the reach and impact of the program [9]. TNP was successfully implemented at 11 VAMC’s, and evaluation of the implementation process identified several barriers to implementation, including competing processes, communication challenges across care settings, need for program buy-in, and recognized need for the program [10–12]. Developing a better understanding of how to sustain care coordination intervientions is vital for planning how to best leverage limited resources ensure patients continue to experience high quality transitions of care [13]. Here we describe plans for sustainment and perceived barriers to sustainment of TNP and comment on their applicability for othere care coordination interventions.

## Methods

TNP enrolled Veterans from 2016 to 2020 at 11 VA medical centers. VAMCs with a high rural Veteran population were the target settings. Recruitment by the TNP team was conducted through outreach to VAMC Directors and medical leadership. Interested VAMCs completed an application and were required to have VAMC leadership support, including a signed letter and memorandum of understanding. Three years of funding was provided through the VA Office of Rural Health to each site to hire a full-time Transitions Nurse, 10% support for a physician site champion, and funds for travel, training and materials to support implementation efforts. Site champions were assigned at each site to be clinical supervisors of the program at their facility. The TNP study protocol has been published [14]. Participating VAMCs made individual decisions about sustaining the program after funding ended. Both effectiveness and implementation outcomes were evaluated in this program and are reported elsewhere [12].

To identify key elements that influenced sustainment of TNP, we conducted a qualitative study to understand plans for sustainment and perceived barriers to sustainment at participating sites. We applied criteria developed by LaPelle et al. [15] to describe various levels of sustainment based on the degree to which TNP continued as originally implemented after TNP funding ended at sites in 2019 and 2020 since implementation start dates were staggered. To assess perceived barriers to sustainment at these 11 sites, we conducted qualitative interviews one year prior to program funding ending (mid-line) and again at the time funding concluded (exit). Mid-line interviews asked specifically about how sites were planning for sustainment at the end of funding. Exit interviews asked about plans for program sustainment, as well as barriers to sustainment. Interviews were conducted with the Transitions Nurse and site champion at each site.

Interview guides (Additional file 1: Appendix 1 and 2) were designed for this study to elicit rich information on perceived sustainability including information on barriers. The interview guides were developed and piloted by the TNP team (CL, HG). Interviews were conducted by trained staff with prior qualitative research experience, including health science specialists (MN, LU), and a PhD trained Anthropologist (CL). All interviewers were female and had limited previous interactions with participants. Interviews were conducted over the telephone between July – August 2018 and March – December 2019 and lasted approximately 20–40 min. All interviews were audio-recorded, transcribed verbatim, and managed in *Atlas.ti* 8.

Interviews were analyzed using inductive-deductive content analysis for themes related to perceptions of sustainability and barriers to sustainment. Initial broad code categories were created based on perceived barriers to sustainment, leadership support, local perceptions of TNP, and program outcomes. Inductive codes were used to identify emergent ideas within these categories and were added throughout coding after discussion by team members. Consensus was reached using a team-based approach [16]. Four analysts (MN, LU, MM, CL) independently coded six transcripts and met to discuss points of divergence and convergence, with regular conversations to discuss emergent codes. Emergent themes were developed through group conversations focusing on patterns in the data and salient information. We also categorized the level of sustainment at each site according to the four levels presented in LaPelle et al. [15]. These include a) no sustainability, b) low sustainability, c) moderate sustainability, and d) high sustainability. Qualitative data are reported according to the Consolidated Criteria for Reporting Qualitative Studies (COREQ) guidelines [16]. This work was funded by VA’s Office of Rural Health and was designated a quality improvement study and was exempt from institutional review board review and deemed not human subjects research.

## Results

We interviewed 11 site champions and 10 transitions nurses from the 11 sites that implemented the TNP. Eight of these interviews were completed one year prior to the end of program funding and thirteen interviews were conducted when funding ended. At the time funding ended, none of the sites planned on sustaining TNP in its original form, but four planned on sustaining some elements. Table 1 summarized the level of planned sustainment at each TNP site at the end of the program.

**Table 1:** Reported Plans for TNP Sustainment after Funding Period Ended for Each Site

Site	TNP Intervention Core Components* Sustained	High Sustainability of Core Components	Moderate Sustainability of Core Components	Low Sustainability of Core Components	No Sustainability of Core Components	Reported Barriers at Site
	Actions taken to implement TNP	Continue under original structure	Continue under alternative organizational structure	Parts of Service continue	Transfer of some or all services to other providers	Barriers that impacted sustainment of core components
**Site 1**	• Assess veteran and family discharge readiness • Follow up appointment with Patient Aligned Care Team (PACT)		• Steps carried out by inpatient coordinators in Utilization Management Department			Leadership & Outcomes
**Site 2**	• Post-discharge phone call to Veteran • Post discharge communication with PACT		• Former Transitions Nurse continues to support TNP Veterans • 10 new care coordinators hired			Leadership & Outcomes
**Site 3**	• Assess Veteran and family discharge readiness • Follow-up appointment with PACT • Post discharge communication with PACT		• Steps carried out by two newly hired nurse transitions coordinators			Leadership, Outcomes, & Role Duplication
**Site 4**	•TNP hospitalist will still take TNP phone calls and offer rural discharge guidance			TNP hospitalist will still take TNP phone calls and offer rural discharge guidance		Leadership & Outcomes
**Site 5**	N/A				Services transferred back to ‘Discharge Expeditor’ role that was in place prior to TNP.	Leadership
**Site 6**	N/A				Transfer of this role to discharge planners including resources and clinic contacts	Leadership, Outcomes, & Role Duplication
**Site 7**	N/A				Services transferred back to process that was in place prior to TNP.	Outcomes
**Site 8**	N/A				Services transferred back to process that was in place prior to TNP.	Outcomes
**Site 9**	N/A				Transfer of this role to discharge planners including resources and clinic contacts	Leadership
**Site 10**	N/A				Transfer of this role to discharge planners including resources and clinic contacts	Outcomes & Leadership

*The core components of the intervention are 1) TN sets up follow up appointment at PACT site 2) TN assesses patient discharge readiness 3) Follow up post-discharge call to patient 4) engage the primary care provider and PACT provider in electronic communication

*Table based on Lapelle’s sustainment work [15]

We identified three themes related to perceived sustainability of TNP. These are 1) Program outcomes that address leadership priorities are necessary for sustainment.; 2) Local perceptions of the need for TNP or redundancy of TNP impacted perceived sustainability; and 3) Lack of leadership buy-in, changing leadership priorities, and leadership turnover were perceived barriers to sustainment. Table 2 defined each theme with an example of a quotation.

**Table 2:** TNP Sustainment Themes & Theme Definitions

Theme	Theme Definition	Example of Theme Quote
*Program outcomes that address leadership priorities are necessary for sustainment*	Leadership is interested in outcomes that illustrate a cost benefit and outcomes that address shortcomings on VA metrics on which facilities are graded.	*“You know, every, like every other hospital in the VA system, the VA, our VA here, our mission statement seems to wax and wane with what’s on the, whatever the issues are of the day with the SAIL report, so when we first started applying for this program, then there were issues with the readmission rate, specifically with certain different special diagnoses and then that transformed into issues with the mortality data, both the 30-day and the in-house mortality.” (site champion).*
*Local perceptions of the need for TNP or redundancy of TNP impacted perceived sustainability*	Some facilities felt they already had services in place that overlapped with the TNP, making the TNP less impactful.	*“It’s very duplicative here […] We are fortunate to have a number of RN coordinators. We have inpatient pharmacists that help with discharge meds, we have PACT nurses that call patients after discharge, we had ortho nurses calling patients after discharge, and so in this setting, this role was quite duplicative and so that was one of our challenges of trying to better understand how we could make it a meaningful intervention, still fulfilling the protocol and not stepping on other providers’ toes..”(Transitions Nurse).*
*Lack of leadership buy-in, changing leadership priorities, and leadership turnover were perceived barriers to sustainment*	In the VA, leadership buy-in in necessary to push facility programs forward. With competing facility priorities, TNP sustainment was not seen as imment at some facilties. When new leardership came on they had to be made aware of TNP and new relationships needed to be develop to create buy-in.	*“Well, I think you certainly have to engage your executive leadership, and I think the most bang for the buck is with your nurse executive because if you get that person that’s a champion for your program, you’re gonna have a lot more chance for success I think when the time comes for budgetary decisions to be made”(site champion).*

*Program outcomes that address leadership priorities are necessary for sustainment.*

Most participants discussed the importance of impactful TNP program outcomes to ensure sustainment. They discussed the feeling that in order to be sustained, TNP needed to impact hospital-wide outcomes such as Strategic Analytics for Improvement and Learning Value Model (SAIL) measures of hospital performance, and metrics from Survey of Health Experience of Veterans (SHEP) which assesses patient satisfaction with hospitalizations. They described that the TNP program needs to be perceived as effective by leadership. Site champions noted that leadership may prioritize different metrics at different times and that leadership priorities may differ by VAMC. However, all participants described that sustainment of TNP was heavily dependent upon outcomes that addressed leadership priorities. One TNP site champion described,

“*You know, every, like every other hospital in the VA system, the VA, our VA here, our mission statement seems to wax and wane with what’s on the, whatever the issues are of the day with the SAIL report, so when we first started applying for this program, then there were issues with the readmission rate, specifically with certain different special diagnoses and then that transformed into issues with the mortality data, both the 30-day and the in-house mortality.”* (site champion).

One site champion commented on his difficulty fitting the TNP into the current hospital priority metrics,

*“Well, the big focus currently for hospital-wide goals would be the SAIL metrics, so the SAIL being the quality and related metrics that go into a composite score that rank VA hospitals has become a major issue because we’ve gone from a five-star to a two-star facility over about the past two years. Two areas that they feel like we’ve gotten worse in or that we could improve on are hospital readmissions and hospital length of stay and hospital mortality and so it appears to me that they’re investing in anything they can think of to improve those metrics. There wasn’t any one specific thing that they said in their emails about these new roles that was addressing this, addressing those issues other than a hope that the TNP or that the, these new transition coordinators can help improve performance on SAIL.”.* (site champion)

Transitions Nurses at some sites felt that leadership priorities shifted based on VA-wide priorities. They described how it was important to continually engage leadership with these priorities in mind.

*“One thing I do have, I have learned is leadership, they are going to focus on what’s priority for the organization at the time”* (Transitions Nurse).

Some Transitions Nurses and site champions felt local TNP outcomes were barriers to sustainment of the program because they did not show a great impact on metrics that facility leadership deemed important. At some sites, there was a lack of apparent effectiveness on outcomes such as hospital re-admissions and emergency department (ED) admissions. One Transitions Nurse summarized this perception,

*“I know we were kind of hoping to have the greater impact on readmission and ED visits but just from looking at the data it just kind of looks like a wash at that point. It doesn’t look like much has been affected that way. I guess it’s kind of hard to look at the quantitative data and say yeah we’re making a huge impact.”* (Transitions Nurse)

Other respondents discussed different types of outcomes that might make a case for sustainment to leadership. A few transitions nurses felt they were able to save time for primary care nurses through their work coordinating care for rural Veterans. One nurse described the challenges that primary care teams face in terms of patient volume and point of contact for patient advocacy. Other nurses described the feedback they received about her role saving primary care nurses’ time. One described,

*“I know that our leaders are very data driven people. But I think we’re also hoping to kind of win them over a little bit with the qualitative side of it, too. And the time saving of Patient Aligned Care Teams (PACT) and other people you know that they then might be able to take on other tasks you know if their time is being freed up”* (Transitions Nurse).

Transitions nurses and site champions also felt that sustainment hinged on financial outcomes. They stated that they would be better equipped to pitch sustainment plans to leadership if they had access to programmatic financial outcomes such as return on investment (ROI), cost savings, and cost effectiveness. One site champion clarified that leadership is interested in quality of care, however financial outcomes of TNP are needed to justify the salary for a full-time nurse position,

*“I think the tension is that it’s pretty obvious that the care is improved when the [TNP nurse] is involved, but it’s been hard to demonstrate financial need when [the TNP nurse] is involved, and I think what the director is primarily interested in. I don’t want to imply that he’s not interested in quality of care, but I think the funding may be the issues that we would ideally show some return on investment.”* (site champion).

Some Transitions Nurses and site champions felt that TNP outcomes did not capture the impact of the program on patient experience and satisfaction. While they did not think that TNP significantly affected hospital-wide outcomes like hospital readmissions and ED visits, they described the importance of Veteran experience and satisfaction. One nurse stated:

*“In the last year for our site we had lots of positive feedback but we don’t really feel like it’s, the data that we’ve gotten back from it has shown us that it’s cost saving so that’s - a worry of ours and how we would present that to just our hospital leadership. And we’re working towards how we can get some cost effectiveness data from you guys in Denver, how we can present it, based on cost and not just on provider and patient satisfaction. And so, I have some great buy-in from all of the providers that I work with and I think from nursing leadership as well, I just don’t know how we’re gonna prove that, from a cost savings perspective .*” (Transitions Nurse)

*Local perceptions of the need for TNP or redundancy of TNP impacted perceived sustainability* Participants described that it is important for TNP to address the local needs at each VAMC and how the program could be adapted to fit those needs. Some sites described the perception that TNP duplicated work that was already occurring in programs and services already in place prior to TNP. At some sites, the Transitions Nurse role overlapped with responsibilities of existing roles like social workers or nurse case managers. Some described the perception that role duplication affected the success of the Transitions Nurse at their site,

*“It’s very duplicative here […] We are fortunate to have a number of RN coordinators. We have inpatient pharmacists that help with discharge meds, we have PACT nurses that call patients after discharge, we had ortho nurses calling patients after discharge, and so in this setting, this role was quite duplicative and so that was one of our challenges of trying to better understand how we could make it a meaningful intervention, still fulfilling the protocol and not stepping on other providers’ toes.”* (Transitions Nurse).

This role duplication was described as a percieved barrier to sustainment, for nurses and champions at these sites did not feel that their colleagues or leadership saw a clear need for the program. Nurses at these sites felt that TNP added something, but there was not a strong local perception that the program was needed,

“*About the only thing different I have added is, you know, of course the PCP communication and the follow-up phone call, and you know, just meeting with the veteran and making sure they understand their diagnosis and their discharge medications and stuff like that”* (Transitions Nurse).

Other sites reported the value of discharge planning and care coordination and described how TNP filled an important gap. They described how in the absence of other care coordination initiatives, TNP prevented rural patients from being “lost,”

*“We have very little care coordination outside of maybe certain surgical specialties and our oncology department, where there’s direct coordination with primary care and to coordinate care for these patients when they leave the acute hospital…[the Transitions nurse] can help tie up a lot of loose ends and prevent things from being dropped or lost in the transition from being hospitalized into acute care, for the tertiary medical center, going back to a permanent care setting”* (site champion).

Many site champions felt that adapting TNP to address various gaps in care at their facilities might make the program more sustainable. They described adaptations that they would make if the program was sustained. One stated:

*“So a lot of what she was doing now, I would love to see her continue to do probably with a slightly expanded patient catchment, so like it could be not only rural patients, and then I think to cover all of the patients that she would need to, we would need probably four of her”* (site champion).

Other site champions mentioned specific groups of patients who would benefit from the TNP intervention,

*“We had hoped or wished we could ask an additional person to do and an example of that might be a number of patients that end up going to skilled nursing facility after discharge were not eligible for the TNP intervention and yet, those are the patients that tend to be at highest risk for errors in their discharge because of the multiple transitions”* (site champion).

Others described what they would do if funding continued,

*“If this program were to be continued, would we expand it to include other different patient populations within the center”* (site champion).

*Lack of leadership buy-in, changing leadership priorities, and leadership turnover were barriers to sustainment.*

Transitions Nurses and site champions at most sites discussed leadership as a barrier to sustainment. Most described the need for leadership buy-In for successful sustainment, but most also described how interactions with leadership demonstrated a lack of investment in the program. One transitions nurse stated,

*“Every once in a while, I’ll be asked, you know, how many patients do you have, but there’s never been any communication I guess as to if people here think it’s working, if it’s not working”* (Transitions Nurse).

Despite the general perception that leadership buy-in was lacking, transitions nurses and champions at most sites felt that buy-in was critical for sustainment. They discussed strategies for leadership engagement, and described how buy-in might affect budgetary decisions. One stated,

*“Well, I think you certainly have to engage your executive leadership, and I think the most bang for the buck is with your nurse executive because if you get that person that’s a champion for your program, you’re gonna have a lot more chance for success I think when the time comes for budgetary decisions to be made”* (site champion).

Several nurses and champions described meetings they had with leadership in order to try to get buy-in for sustainment, but their intereactions ended up showing a lack of buy-in,

*“I’ve had informal discussions with the head of our service line about how the project is going and whether this project is something they would want to try to fund after ORH funding expires, but that’s probably the extent of my discussions with other people. There are, there was a nursing research day about a month ago, and our nurse put together a small poster for that, and she mentioned, you know, the data from the program suggests that there is a decrease 30-day mortality risk …for the people… enrolled in the TNP program, versus their comparable controls, and she was very interested in hearing more about that”* (Transitions Nurse).

However, despite plans to make leadership engagement ongoing and effective, most sites reported difficulty in maintaining leadership engagement,

*“When we first started we had to get approval from the chief of staff and the chief nurse to apply for the program …and he would intermittently ask me how it was going, but we haven’t had significant day-to-day leadership involvement with the program.”* (site champion)

Some Transition Nurses described their perception that ongoing leadership engagement was necessary for sustainment. One said,

*“Don’t wait until like the last six months, you know, before the grant is about to run out, you know. You need to be out there pretty much the entire time, you know, speaking the language and trialing and doing various things and keeping them [leadership] updated, you know, about the success of the program”* (Transitions Nurse).

Interestingly, sites that felt they had leadership support did not feel that this would translate to sustainment of the Transitions Nurse position.

*“We had been receiving a lot of promises, or interest, from site leadership, including different levels of executives who would offer us support, but offering support and actually putting [a position request] request in to the FTE (full time equivalent) Committee are two totally different things”* (site champion).

At other sites, Transitions Nurses and site champions described leadership turnover as a barrier to sustainment. One described,

*“I think the original leadership that approved this has turned over 100%, our nursing lead and our chief of staff lead, they’re all different than when this was originally proposed”* (site champion).

Another important factor playing into the need for leadership buy-in to achieve sustainment is the unpredictable nature of leadership turnover. Participants described that enthusatic buy-in from current leadership does not guarantee that several years later the same support and approval will be there. Site champions discussed how confusion caused by leadership turnover made it difficult to plan for transitions nurse position after funding ended.

*“We spent a lot of time working with the Manager of the Care Coordination world, [name], who just really liked working with [the Transitions nurse], really wanted her in her department, kept hinting that things were in motion, and then she had a boss named [name], who we could never connect with, but then [she] got moved off, so we went to [name], the chief nurse, and he supported us as well, but he couldn’t help us with an [position] creation and then we were waiting to meet with the deputy chief of staff, and we did, and they said, sounds great, makes sense to keep it going. We’ll just do that. And then, unfortunately, our executive left, our chief of staff left, […], so it just stalled, and so we never really knew who was the decider”* (Transitions Nurse).

One site champion notes how it is difficult for a new leader to grasp the importance of TNP when they are trying to learn an entire new facility:

*“No. I think that they don’t know anything about it and that’s a very honest answer. I think with the transitions. We literally have had a chief of staff for a little less than a year who is getting his head around any number of hundreds of things, [….]they don’t really know about it. I think and our chief nurse, I think that there was, our chief nurse, I don’t even know if she was ever involved with prior conversations with our prior nurse, but there wasn’t, per my nurse, there wasn’t a lot of nursing support for his role, but I don’t know if that’s accurate or not, but I don’t think they’ve had the opportunity to really learn about this, again, totally different team, getting up to speed on the institution in general”* (site champion).

## Discussion

The goal of this study was to better understand the factors affecting perceived sustainability of TNP. We found that five TNP sites planned to sustain some aspects of the program, and seven sites had no plans for sustainment. We built on previous work that shows that organizational complexities impact sustainment on a local level and identified three themes across 11 sites related to sustainability that are likely relevant for other care coordination interventions [4].

Our findings can be interpreted in the context of similar programs, such as the IMPACT care coordination intervention. IMPACT aimed to reach older adults with major depressive disorder through a collaborative care intervention in primary care. IMPACT defined sustainment as all or parts of the program continuing after funding ended. Sustainment of IMPACT depended on the organizational support, the availability of staff trained in the intervention, and funding. Much like TNP sustainment was influenced by leadership priorities concerning care coordination needs, IMPACT sustainment was dependent on organizational support of collaborative care models [13].. Also like IMPACT, TNP sustainment encountered barriers to continued funding for staff trained in the intervention. Importantly, in TNP the primary barrier to funding was the ability to show economic outcomes that justified spending money on the intervention [12]. To address the financial barrier to sustainment, other care coordination programs, like the VHA C-TRAC program, include locally targeted financial cases as part of their sustainment process. C-TRAC begins thefinancial case process soon after implementation, and program results are reported to leadership and other relativent stakeholder [17].

Transitions nurses and site champions overwhelmingly stated that program outcomes were necessary for program sustainment. However, they felt that the “right” outcomes might change with leadership priorities and felt that financial outcomes might be most critical for sustainment. This reflects previous work that shows newly disseminated clinical programs must be valued within the healthcare systems they are used [18] and that program success is critical for sustainment [8]. Unfortunately, without data to show reduced readmissions or cost savings to the facility, justification was not sufficient for leadership to fund the nurse position, even though Veterans reported high satisfaction with their care. A positive impact on quality metrics can help program sustainment. However, quality metrics are weighted and priorities shift over time. This can have implications for where funding flows and can create real challenges for program sustainment. Changes in leadership priorities should be addressed in pre-implementation planning. Priorities will constantly change, so it is important to collect data that can address a number of outcomes to illustrate the impact of a program in different ways.

Leadership turnover was a perceived barrier to sustainment of TNP at several VAMCs. Enthusiastic buy-in from current leadership does not guarantee that several years later the same support and approval will be there. VAMC leadership approval was required to receive program funds. However, leadership engagement tapered off at the majority of sites as the program progressed. Weiner’s organizational theory of innovation implementation suggests that leaders are critical in creating readiness for change, ensuring innovation-values fit, and developing plans, practices, structures, and strategies to support implementation [19]. Building a program that is adaptable and continually engages leaders to understand program impact is likely a key element of sustainability. Training implementers and site champions to engage with leadership may help address barriers related to leadership turnover and engagement. In the final year of TNP, we offered an in-person training on building presentation skills and executive presence with standardized actors. All nurses and site champions prepared brief pitches to support leadership engagement. Building these skills earlier in implementation may have helped keep leadership engaged in conversations about adapting TNP to local needs and to secure funding for the Transition Nurse position. In subsequent cohorts, the TNP position funding has been reduced to a partial position so that the sites make the decision to participate and to financially support a majority of the position for the Transitions Nurse at the time of implementation. Future work will evaluate whether this change influences sustainability.

Some of the Transitions Nurses felt that TNP duplicated some work already occurring. This has important implications for sustainment, as previous work shows that in order to be valued, programs also need to be sensitive and adaptable to the new system’s pre-existing context, resources and locally defined goals [5]. A thorough needs assessment should be conducted at each site pre-implementation to become attuned to the unique needs and culture of each site [20]. Pre-implementation interviews should be conducted to gather cultural context components and build relationships with key stakeholders who may influence program sustainment [21]. Understanding what these stakeholders value can help inform evaluation design and program outcomes [22]. Further, understanding the role of key stakeholders may reduce perceptions of role duplication [23]. The TNP team conducted in-depth pre-implementation assessments [11, 14, 15] and identified concerns about role duplication early on. During the implementation stage, facilitation from the TNP team focused on how to fit the TNP intervention into existing processes to avoid role duplication. Despite these efforts, transitions nurses were not able to avoid duplicating key elements of the patient discharge process at some sites. This indicates that greater flexibility with the intervention, and who completes each TNP core components may be necessary. The Coordinated Transitional Care (C-Trac) program similarly found that it was important to integrate into existing discharge processes rather than replicating them [5]. In an empirical review of the current sustainment literature, Stirman found that the fit of the program or intervention with the system or organization and the degree to which the intervention or program could be modified were crucial to sustainment [24].

The majority of transitions nurses and site champions expressed that TNP filled important gaps in care at their sites but did not feel that program outcomes fully communicated the impact of their work. While the TNP evaluation was responsive to site requests and suggestions, it was not always possible to communicate program impacts in a way that satisfied all stakeholders. As TNP continues to scale up, future work will assess the feasibility of collaborating with site leadership and Transtion Nurses to design program outcomes that meet the needs of organizational decision makers.

### Limitations

This is a qualitative study in the VA. Our findings may not be generalizable to other healthcare systems. Other limitations include the modest number of interviews conducted, although the multiple sites represented were diverse in size and geographic location, and the consistency of findings across sites suggests identified themes are valid. The fact that some of the interviews were conducted one year prior to funding ending and the lack of data on final sustainment outcomes is also a limitation.

## Conclusions

Our findings provide insights for care coordination interventions planning for sustainment. These include conducting a thorough needs assessment pre-implementation and ongoing engagement with leadership to determine what they need to see to justify a nursing position. Assessing actual sustainment should be addressed in future work.

## Supplementary Information

**Additional file 1: Appendix 1.** Exit Interview Guide for Transitions Nurse for TNP. **Appendix 2.** Exit Interview Guide for Site Champion TNP

## Data Availability

Interview transcripts are not available for privacy reasons. It will take significant effort by the authors to further de-identify the raw qualitative data and we do not have the resources. Therefore, we will not share raw qualitative data. Due to ethical restrictions, we are unable to share data publicly because the data contains potentially identifying and/or sensitive patient information.

## Abbreviations

IRB: Institutional Review Board
IMPACT: Inter Multi-Disciplinary Prescreening Assessment Crisis Team
PACT: Patient Aligned Care Team
RN: Registered Nurse
PCP: Primary Care Provider
SAIL: Strategic Analytics for Improvement and Learning Value Model
SHEP: Survey that asks a random sample of recently discharged patients about important aspects of their hospital experience
ROI: Return on Investment
ED: Emergency Department
FTE: Full Time Equivalent
ORH: Office of Rural Health
C-Trac: Coordinated Transitional Care Program
TNP: Transitions Nurse Program
TN: Transitions Nurse
VA: Department of Veterans Affairs
VAMC: VA Medical Center
