# Supplementary material for: Sustainment stories: a qualitative analysis of barriers to sustainment of the National Rural Transitions of Care Nurse Program
Source: BMC Health Serv Res. 2022 Jan 28;22:119. doi: 10.1186/s12913-021-07420-1 (PMC8796421; doi:10.1186/s12913-021-07420-1)
Supplement: Supplementary file 1 — Additional file 1: Appendix 1. Exit Interview Guide for Transitions Nurse for TNP. Appendix 2. Exit Interview Guide for Site Champion TNP [file 12913_2021_7420_MOESM1_ESM.docx]

**Appendices**

**Appendix 1 - Exit Interview Guide for Transitions Nurse for TNP**

Intro Script: Thank you for taking the time to talk with me today. We are very grateful for your time working with TNP and are sorry to see you leave. I would like to ask you some questions about your experience as a transitions nurse.

This interview is completely voluntary, please feel free to stop at any time or to skip a question you would rather not answer. The information you give us will be used only by the TNP team, will not be shared with site champions or other TN’s, and will be reported in aggregate in future work. I would like to record this interview. Would that be ok with you?

***Grounded prompts: If responses are limited or require clarification, probes may be used to illicit more detailed responses. Probes should use words or phrases presented by the participant using one of the following formats:***

1. ***1. What do you mean by ____________?***
2. ***2. Tell me more about ____________.***
3. ***3. Give me an example of ____________.***
4. ***4. Tell me about a time when ____________.***
5. ***5. Who __________?***
6. ***6. When __________?***

1. What circumstances prompted you to start looking for another job?

2. Is there anything that could have kept you in the TN role?

**Next, I would like to ask some questions about your experience with the TNP:**

3. What was the best part of the TN role?

4. Were there any parts of the TN role you disliked?

- 1. *[If so] How can the TNP team improve the role?*

5. Were there any parts of the TN role that did not benefit Veterans?

a*. How can the TNP team adapt the program to be more Veteran centric?*

6. Do you feel you had the necessary training to be successful in your role?

1. *[If not] How could it have been better?*

7. Did you feel you had the tools, resources, and guidance to be successful in your role?

1. *[If not] Which areas could be improved and how?*

8. Did your role change since you were hired, and if so, in what ways?

9. Can you tell me how your contributions to the organization and to Veterans were recognized?

1. *(If they don’t mention, ask about TNP team vs. hospital/PACT sites they work with)*

10. Can you tell me about your relationship with your site champion?

***Next, I would like to ask a few questions about your plans to sustain TNP program at your site.***

1. Tell me about your experience working towards sustainment of TNP and creating such a long-lasting impact at your site?
   1. Probe: What specific factors contributed to the success of TNP at your site?
2. What lessons have you learned that could be shared with other TNs/champions in the program?
   1. Probe: what to do and what not to do when planning the long-term sustainment of the program?

***Next, I would like to ask you a few questions as you prepare to leave the program.***

1. Is anyone assigned to work on the transitional needs of rural Veterans?
2. Have you notified your colleagues of the end of your role? If so, how?
3. If applicable, have you introduced your replacement to your contacts internally (rounding teams, etc.) or externally (rural PACT, home care, etc.)? If so how, (email, in person, phone)?
4. If applicable, have you trained anyone in the intensive care coordination TNP intervention (the 4 core components?) If so, whom and how?
5. Who have you identified as your TNP contact if Veterans or providers have questions after your departure?

***Next, I would like to ask you a few questions about your process of delivering the TNP intervention. We sent you a process map from the midline evaluation, back in December of 2017.***

1. Have there been any changes to the process as shown on the map?
   1. Probe: are there any process steps that you no longer do? Are there any new process steps?
   2. Probe: has the sequence of these steps changed?
   3. Probe: have there been any new roles you worked in this process?
   4. Probe: have there been any additional bottlenecks/issues in your process?
   5. Probe: is the time estimation accurate for these steps?
   6. Probe: did you have to wait on anything in between the steps?

***Thank you for that information. Next, I have a few more questions about changes in the TNP program at your site.***

1. Have there been any changes to the TNP program or how you delivered the intervention at your site? In the past, we tracked changes such us modifying exclusion/inclusion criteria, opening the program to additional services/teams, etc. Can you think of any other changes that took place in the last year and a half?

***If yes – ask to describe the change and continue with questions below (2-8). If more than one change took place, ask to describe them all and probe on the one that seems most interesting/most profound.***

***If no – skip to question 9.***

1. WHAT Part 1: WHAT component or part of the intervention was changed in this adaptation; in other words, what was the nature of the change?

PROBE: For instance, was it a change to program content, format, delivery mode, staff delivering it, patients eligible, where, when or how it was delivered, or what?

1. WHAT Part 2: How would you describe the *type* of change involved in this adaptation?

PROBE: Specifically, what did the change involve? Was something added, deleted, changed to better fit the patients, delivered at a different time or in a different way?

1. WHO was responsible for first suggesting or initiating this change?

PROBE: Was this the person or persons the ones who implemented the change? (If not, who implemented the adaptation?).

1. WHEN during the TNP program was this adaptation first made?

PROBE: If early and if not clear, probe if was before or after began implementing.

1. HOW or on what BASIS was this change made?

PROBE: based on challenges implementing, on time concerns, on results or data you collected, on external or administrative concerns, feedback from patients or staff, or what basis?

1. WHY Part 1: WHY was this adaptation made?

PROBE: For example, to get more people to participate, to make the program attractive to more settings, to increase its effectiveness, to make it easier to deliver, to make it easier to maintain or reduce costs, etc.?

WHY Part 2: 6a. Was this adaptation a result of EXTERNAL factors or INTERNAL issues?

PROBE: EXTERNAL factors (for example change in organizational policies, reimbursement changes) or INTERNAL issues (such as workflow, changes in staff or similar issues)?

1. What was the short-term IMPACT of this adaptation?

PROBE: Did it have highly visible results? For example, did it result in more or less participation by patients, get more or fewer settings or staff involved, improve or decrease consistency of delivery, improve or reduce outcomes, reduce or increase time or costs? We understand that you may not have concrete outcomes results at this time – please tell us your best perception of the impact of this adaptation thus far.

1. Is there anything else about the TN role you would like to share?
2. Is there anything else about your experience in the TNP you would like to share?

***Again, thank you so much for participating in this interview. We appreciate your time and responses. We also appreciate your work in implementing TNP at your site. We wish you the best in your next role.***

**Appendix 2 = Exit Interview Guide for Site Champion TNP**

Intro Script: Thank you for taking the time to talk with me today. We are very grateful for your time working with TNP, and are sorry to see you leave. I would like to ask you some questions about your experience with TNP.

This interview is completely voluntary, please feel free to stop at any time or to skip a question you would rather not answer. The information you give us will be used only by the TNP team and will be reported in aggregate in future work. I would like to record this interview. Would that be ok with you?

***Grounded prompts: If responses are limited or require clarification, probes may be used to illicit more detailed responses. Probes should use words or phrases presented by the participant using one of the following formats:***

1. ***1. What do you mean by ____________?***
2. ***2. Tell me more about ____________.***
3. ***3. Give me an example of ____________.***
4. ***4. Tell me about a time when ____________.***
5. ***5. Who __________?***
6. ***6. When __________?***

1. What circumstances prompted your site to leave TNP?

2. Is there anything that could have kept TNP at your site?

**Next, I would like to ask some questions about your experience with the TNP:**

3. What was the best part of being a TNP site champion?

4. Were there any parts of the TNP site champion role you disliked?

- 1. *[If so] How can the TNP team improve the champion role?*

5. Can you tell me your perception of TNP’s contributions to the organization and Veterans?

*6.* Were there any parts of TNP that did not benefit your organization or Veterans?

a*. How can the TNP team adapt the program to be more Veteran centric?*

7. Do you feel you, as a champion had the necessary training to be successful in your role?

8. Do you feel like the training in Denver was adequate to implement TNP?

1. *[If not] How could it have been better?*

9. Did you feel you had the tools, resources, guidance and Leadership support for TNP to be successful?

*[If not] Which areas could be improved and how?*

10. Can you tell me about your relationship with the TN at your site?

***Next, I would like to ask a few questions about your plans to sustain TNP program at your site.***

1. Tell me about your experience working towards sustainment of TNP.
   1. Probe: What contributed to creating such a long-lasting impact at your site? **OR**
   2. Probe: What specific factors contributed to the success of TNP at your site?
2. What lessons have you learned that could be shared with other TNs/champions in the program?
   1. Probe: what to do and what not to do when planning the long-term sustainment of the program?

***Next, I would like to ask you a few questions as you prepare to leave the program.***

1. Is anyone assigned to work on the transitional needs of rural Veterans?
2. Have you notified your colleagues of the end of your role? If so, how?
3. If applicable, have you introduced your replacement to your contacts internally (rounding teams, etc.) or externally (rural PACT, home care, etc.)? If so how, (email, in person, phone)?
4. If applicable, have you trained anyone in the intensive care coordination TNP intervention (the 4 core components?) If so, whom and how?
5. Who have you identified as your TNP contact if Veterans or providers have questions after your departure?

11. Is there anything else about TNP you would like to share?
